# Supplementary material for: Does the openness of the Boundary Shell system influence the sustainable development of the high-tech industry?
Source: PLoS One. 2024 Feb 16;19(2):e0298180. doi: 10.1371/journal.pone.0298180 (PMC10871513; doi:10.1371/journal.pone.0298180)
Supplement: Attachment — Submitted filename: Response to Reviewers 2024.01.06.docx [file pone.0298180.s001.docx]

**RESPONSES TO REVIEWERS’ COMMENTS ON THE SUBMISSION**

“**Does the openness of the Boundary Shell system influence the sustainable development of the high-tech industry?**”

(Manuscript ID#: PONE-D-23-29413)

We are very grateful to the Editor-in-chief, Associate Editor and anonymous reviewers for offering constructive comments on our submission and giving us the opportunities to revise and resubmit this manuscript. In this revised version, we have made substantial revisions to the manuscript in response to the reviewers’ constructive comments. Thanks to your suggestions, the content and presentation of our article have been significantly improved.

Our response displays the comments made by the editorial team in blue font. All modified or added words in the revised version are highlighted in red font.Please find below our point-to-point response to two anonymous reviewers.

**RESPONSE TO REVIEWER 1**

**Question 1:**

There is an incorrect citation in line 120, and the font inconsistency in line 163.

**Response 1:**

We appreciate your comments and suggestions.

Thank you for bringing to our attention the issue of an incorrect citation in line 120 and a font inconsistency in line 163. We have reviewed the cited material and made the necessary corrections to ensure its accuracy. Additionally, We have addressed the font inconsistency in line 163 by applying consistent formatting throughout the document. Your meticulous attention to detail is greatly appreciated as it helps us maintain the highest standards of academic integrity.

**Question 2:**

The introduction of the paper does not effectively introduce the topic and feels abrupt. It is recommended to include some background information in the introduction, such as the importance of the high-tech industry and the significance of sustainable development, to provide a better foundation for the subsequent discussion.

**Response 2:**

Thank you for your insightful comments on our manuscript. We appreciate your suggestion to improve the introduction of our paper.

We agree that the current introduction does not effectively introduce the topic and feels abrupt. To address this issue, we have revised the introduction to include more background information on the importance of the high-tech industry and the significance of sustainable development. The examples of modifications in manuscript are as follows (indicated in italics):

（1）**The importance of the high-tech industry**

*With the rapid development of technology, there has been a huge leap in the scale and quality of manufacturing, and economic development has shifted from high-speed growth to a stage of high-quality development.Along this line,high-tech industries, as engines of economic growth and innovation, have prominent characteristics such as high technical intensity, heavy investment in research and development, wide adoption, and low energy consumption, high-tech industrie play a crucial role in upgrading the national industrial structure and changing the economic growth model. High-tech industries promote economic growth and development by introducing new technologies, products, and services, and improving production efficiency. In terms of automation and intelligent technology, which are more prominent features of high-tech industries, their advantages in improving production efficiency, reducing resource waste, and environmental pollution are even more obvious. In addition, with the continuous improvement of high-tech industrialization, they provide new types of jobs, create new employment opportunities, and promote social development and stability*

**（2）The significance of sustainable development**

*In recent years, we are faced with a multitude of complex challenges that require a comprehensive and integrated approach.Sustainable development is a holistic approach to the complex challenges facing our world today, with its core being the pursuit of balance between economic growth, social progress, and environmental protection. As populations grow and economies develop, we are confronted with daunting challenges such as energy scarcity, environmental pollution, and climate change. Only through sustainable development can we achieve the rational use of resources and the protection of our environment. In this digital age, businesses must constantly adapt to changing environmental and societal demands. By adopting sustainable business models and technologies, high-tech industries can reduce resource waste, lower costs, enhance their competitiveness and innovation capabilities. Sustainable development presents new market opportunities for high-tech industries, driving sustained economic growth. By embracing sustainable practices, high-technology industries can reduce their negative effects on the environment and society while promoting sustainable economic growth and technological innovation.*

The complete set of modifications can be found by referring to page2-3 of the manuscript.We hope that this revision addresses your concerns and improves the overall quality of our manuscript. Thank you again for your valuable feedback.

**Question 3:**

The theoretical framework section lacks detailed exposition and explanation. It is suggested to delve deeper into the relationship between the Boundary Shell system's openness and sustainable development, and provide a clearer theoretical framework and analytical model.

**Response 3:**

Thank you for your constructive comments on our manuscript. We appreciate your suggestion to improve the theoretical framework section.We agree that the current theoretical framework section lacks detailed exposition and explanation. To address this issue, we have revised the section to delve deeper into the relationship between the Boundary Shell system's openness and sustainable development.The examples of modifications in manuscript are as follows(indicated in italics):

（1）**The relationship between the Boundary Shell system's openness and sustainable development**

*The Boundary Shell theory is a theory proposed by Professor Cao Hongxing to study the general laws of system periphery. It emphasizes that the interaction between the system and its environment is carried out through the boundary. In this theory, the boundary is not only the external limit of the system, but also the key area for the interaction between the system and the environment. In the Boundary Shell system, openness is an important concept. Openness refers to the ability of the system to effectively exchange materials, energy, and information with its environment. This openness enables the system to obtain necessary resources from the environment, as well as discharge waste into the environment, thus achieving the normal operation and development of the system.However, openness does not mean unlimited exchange. Excessive openness may lead to excessive consumption of resources, environmental damage, or even system collapse. Therefore, how to achieve sustainable development of the system while maintaining openness is an important issue facing the Boundary Shell system.According to the Boundary Shell theory, the key to solving this problem lies in finding a balance. This balance should not only ensure that the system can obtain necessary resources from the environment, but also protect the environment from excessive development and destruction.*

*In the perspective of sustainable development, the Boundary Shell theory provides a brand-new way of thinking. By maintaining the security and stability of the system while ensuring interaction and openness with the environment, the Boundary Shell theory contributes to achieving sustainable development of the system. Specifically, by controlling the influx of substances, energy, and information, the Boundary Shell can prevent the system from being disturbed too much, protecting its core values and resources. At the same time, through open boundary gates, the system can obtain necessary resources and information to support its continuous development. Therefore, we can say that the openness and security of the Boundary Shell system are important conditions for achieving sustainable development.*

The complete set of modifications can be found by referring to page3-5 of the manuscript.

**（2）The supplementary to the theoretical framework.**

Examples of Boundary Shell phenomena can be found in both natural and human systems, such as biological membranes, national borders, atmospheric layers, livestock pens, eggshells, clothing, and internet firewalls, we have provided a more comprehensive analysis of the underlying theories and concepts that support our research, including the concept of open systems theory and its application to the Boundary Shell system. We have also developed a clearer theoretical framework that illustrates the complex interplay between the system's openness and sustainable development,as shown in Fig 1.

**Fig 1.A Schematic diagram of the bound-shell structure.**

The complete set of modifications can be found by referring to page4-5 of the manuscript.

**（3）The supplementary to analytical model**

In light of the constructive feedback provided by the reviewers, we have taken into account their suggestions and made significant enhancements to our analytical model. By adopting an openness-centric approach from the boundary shell system, we have developed a groundbreaking analytical framework aimed at analyzing and ascertaining stability within the boundary shell system. This innovative model is depicted in Figure 2, which showcases its intricate design and functionality.

**Fig 2.Analysis flowchart of upper and lower threshold analysis for Boundary Shell system.**

The complete set of modifications can be found by referring to page 9 of the manuscript.

We believe that these revisions will enhance the clarity and depth of our theoretical framework, and provide a stronger foundation for the subsequent analysis in our paper. Thank you again for your valuable feedback.

**Question 4:**

The description of the research methods is too brief and does not provide specific research steps and data sources. It is recommended to provide a detailed description of the research methods, including the data collection and processing process, model establishment and validation process, etc., to help readers better understand and evaluate the reliability of the research.

**Response 4:**

Thank you for taking the time to review our manuscript and provide valuable feedback. We appreciate your suggestion regarding the brief description of our research methods.We understand that a detailed description of our research methods is crucial to help readers better understand and evaluate the reliability of our study. Therefore, we have revised our manuscript to include a more comprehensive explanation of our research methods, including the data collection and processing process, model establishment and validation process.

（1）**The data collection**

We have carefully read through the comments and made revisions to the data collection section of our manuscript.We have provided a more detailed description of the data sources, including the time frame of data collection, the criteria and methods used for sample selection. Additionally, we have also described in greater detail the data processing methods employed to ensure the reliability and validity of the data.The examples of modifications in manuscript are as follows(indicated in italics):

*In this present study, we have employed a bibliometric methodology, drawing upon the comprehensive database of citexs as our foundation. Leveraging the analytical platform of Citexs, we embarked on a meticulous exploration of the extensive corpus of literature to uncover valuable insights. By focusing our keyword search on high-tech industries, we identified a total of 3962 articles published between January 2013 and December 2023, with an average annual publication rate of 361 articles. As illustrated in Fig 3, the pinnacle of annual publication volume was reached in 2021 at 467 articles, while the most rapid growth rate was observed in 2020 at 25.65%. These findings indicate that research within this domain has experienced a remarkable acceleration, currently positioned in a phase of ascending momentum.*

**Fig 3. Annual publication trends of related literature from 2013-01 to 2023-12 high-tech industries**

*Utilizing the analytical platform of citexs, a bibliometric analysis revealed that between January 2013 and December 2023, the top 20 authors in terms of publication output within the field of high-tech industries research were identified.To ensure the reliability of data acquisition, the scope of industry technology is primarily determined by delving into the realms of industry reports, technical documents, relevant literature, and esteemed industry standards pertaining to the target sector. Subsequently, in alignment with the lexicon of high-tech industries, a search expression is formulated, enabling the selection of a database and the acquisition of initial metrics.*

*In order to ensure the availability and consistency of data, this study has implemented a deliberate sampling approach, focusing on a specific group of 30 provincial-level administrative regions in the People's Republic of China, during the period from 2011 to 2021. This cohort has been selected while excluding Hong Kong, Macao, Taiwan, and Tibet. The data sources for this study primarily consist of official publications, ensuring the reliability and accuracy of the data utilized in the analysis. By adopting this sampling strategy, the study aims to minimize potential errors and inconsistencies, and provide robust and credible findings. including the "China Statistical Yearbook", "China Population and Employment Statistical Yearbook", "China Energy Statistical Yearbook", "China Environmental Statistical Yearbook", "China Science and Technology Statistical Yearbook", as well as provincial statistical yearbooks. Due to the one-year suspension of publication of the "China High-tech Industry Statistical Yearbook" in 2018, Due to the unavailability of statistical data for the year 2017, the collection of statistical data was delayed by a year. The "China High-tech Industry Statistical Yearbook" has already provided specific statistical data on the proportion of high-tech industry export value to manufacturing industry export value. Therefore, no further data processing is required for this data. This will ensure that the analysis is conducted using the most recent and reliable data available, providing accurate and meaningful results.*

*The complete set of modifications can be found by referring to page 9-12 of the manuscript.*

（2）**Processing process**

We have carefully perused your revision suggestions and have relocated the processing process to the methodology section of our research, in order to facilitate a more comprehensive understanding and evaluation of the reliability of our study by the readers.The text details the process of standardizing collected data through the utilization of range normalization methods.The examples of modifications in manuscript are as follows(indicated in italics):

*This is done to eliminate any potential impact of different unit dimensions that may exist within the data. By using an indicator range of [0,1], the collected data is standardized in a manner that ensures comparability and consistency across all units of measurement. The standardization formula is also provided to ensure uniformity and accuracy in the calculation process. By employing this approach, the data is transformed into a format that is more conducive to analysis, allowing for meaningful insights to be drawn from the results.*

*(1)*

*The CRITIC method is a powerful multi-criteria decision analysis technique that enables the determination of the relative significance of various criteria. Utilizing the Pearson correlation coefficient, the method computes the intercorrelations among criteria, which serve as a foundation for the calculation of criterion weights. Furthermore, the approach acknowledges the exclusiveness and interdependence between criteria, thereby enhancing its ability to capture the intricacies of the decision-making environment.*

$\text{i}=\text{1,2,3,...,m,j}=\text{1,2,3,...,n}$ *(2)*

$\text{i}=\text{1,2,3,...,m}$ *(3)*

The complete set of modifications can be found by referring to page13-14 of the manuscript.

**（3）Model establishment**

We have carefully perused your revision suggestions and have relocated the model establishment to the methodology section of our research, in order to facilitate a more comprehensive understanding and evaluation of the reliability of our study by the readers.

In response to the reviewer's comments, we have taken into considerationIn response to the reviewer's comments.

In the methodology section, we have incorporated a research model consisting of three components: the Upper limit model of openness of Boundary Shell system, which is based on the reaction-diffusion equation; the Lower limit model of openness of Boundary Shell system, which discusses the entropy equilibrium of the system in accordance with the second law of thermodynamics; and the revised Boundary system ratio model of Boundary Shell system. By doing so, we aim to provide a comprehensive understanding of the complexities inherent in the study of Boundary Shell systems. The Upper limit model serves as a cornerstone for exploring the upper bounds of openness within these systems, while the Lower limit model sheds light on the lower limits that govern their behavior. Furthermore, the refined Boundary system ratio model offers a nuanced perspective on the interplay between various factors that influence the dynamics of Boundary Shell systems.Three parts of the model are as follows：

① Upper limit model of openness of Boundary Shell system

② Lower limit model of openness of Boundary Shell system

③ Boundary system ratio model of Boundary Shell system

The complete set of modifications can be found by referring to page14-16 of the manuscript.

**（4）Validation process**

Regarding your comment about insufficient research process, we understand your concern. In order to address this issue, We have reorganized the "Comparison of the model before and after refinement" section in our study to compare our current findings with traditional models.This is to verify the effectiveness and limitations of the model.As illustrated in Figure 9.

A comparative analysis of the two sets of indicator values before and after modification is presented within the same chart. It can be observed that the innovation capability boundary system prior to modification was relatively unstable during this period, resulting in uncertainty and risk in enterprise innovation activities. However, following model modification, the fluctuation amplitude of all indicators has significantly decreased, thereby effectively controlling their volatility. Furthermore, the stability and reliability of the innovation capability boundary system have been notably enhanced, reducing the uncertainty and risk associated with enterprise innovation activities. This out come substantiates the effectiveness of the proposed methodology.The integration of indicator weights into the refined boundary system ratio model affords a more exhaustive and unprejudiced appraisal of the sustainable development capabilities of high-tech industries, relative to the original model.

The complete set of modifications can be found by referring to page 24-25 of the manuscript.

We believe that these revisions will greatly enhance the clarity and transparency of our research methods, and we hope that they will improve the overall quality of our manuscript. Thank you again for your constructive feedback and suggestions. We look forward to hearing from you soon.

**Question 5:**

The description of the results analysis is not clear, and the interpretation and explanation of the charts and data are insufficient. It is suggested to add detailed explanations and analysis of the charts and data in the results analysis section to better support the conclusions and arguments.

**Response 5:**

Thank you for your constructive feedback and suggestions for improvement. We appreciate your time and effort in reviewing our manuscript.

We have carefully considered your comment regarding the clarity of the results analysis and insufficient interpretation and explanation of the charts and data. In response to your suggestion, We have redrawn clear charts for the data and added detailed explanations and analysis of the charts and data in the results analysis section. This includes a thorough description of the methodology used to collect and analyze the data, as well as a clear and concise interpretation of the findings.We have also provided additional context and background information to help readers better understand the significance of the results and how they support our conclusions and arguments.To further enhance the clarity of our results analysis, we have revised the language and structure of the text to ensure that it is more accessible to a wider audience.

The complete set of modifications can be found by referring to page 19-24 of the manuscript.The revised figure is as follows:

**Fig5. The ratio of C1- C3 in 2011-2022**

**Fig6. Ratio of bounds between D1 and D2 from 2011 to 2022**

***Fig.7 Trend change before improvement in Boundary System***

***Fig.8 Trend change after improvement in Boundary System***

We believe that these revisions address your concerns regarding the clarity of the results analysis and provide a more comprehensive and robust presentation of our findings. We hope that you will find these changes satisfactory.

Thank you again for your constructive feedback and suggestions.

**Question 6:**

The conclusion is too brief and does not effectively summarize the main points and highlights of the paper. It is recommended to provide a more detailed summary of the research findings, indicating their significance and contributions, while also discussing future research directions and value.

**Response 6:**

Thank you for your insightful comments and suggestions for improvement. We have carefully considered your feedback regarding the brevity of the conclusion and its inability to effectively summarize the main points and highlights of our paper. In response to your suggestion, we have made the following revisions:

① We have expanded the conclusion section to provide a more detailed summary of our research findings, including a clear indication of their significance and contributions to the field.

② We have also incorporated a discussion on future research directions and potential implications of our findings, highlighting the value and relevance of our work to the broader scientific community.

The examples of modifications in manuscript are as follows(indicated in italics):

# *5.Conclusions remarks and implication*

*5.1 Conclusions*

*Through a series of investigations conducted in this paper, we aim to explore whether the openness of Boundary Shell systems impacts the sustainable development of high-tech industries. Throughout this process, relevant data has been collected and subjected to meticulous analysis. In this section, we will provide a comprehensive summary and discussion of our research findings with the purpose of gaining a deeper understanding of the factors and underlying mechanisms that drive the sustainable development of high-tech industries through the lens of Boundary Shell theory. The main results are summarized as follows:*

(1) *This study delves into the complex interplay between the quantity of Boundary Shell and internal system.*

*(2)After the refinement of the boundary system ratio model, a significant shift in the prioritization of indicator industries has occurred.*

*(3)The boundary system ratio model has undergone a continuous refinement process to enhance its capacity in evaluating the sustainable development capabilities of high-tech industries.*

*5.2 Policy implication*

*In light of the aforementioned discoveries, the following research implications are suggested.*

*(1) The principle of entropy equilibrium provides a valuable framework for evaluating the stability and lifespan of high-tech industry Boundary Shell systems.*

*(2) The openness of Boundary Shell systems plays a critical role in promoting the sustainable development of high-tech industries.*

*(3)Collaboration is the key to unlocking the full potential of high-tech industries and ensuring their long-term success.*

*5.3. Limitations*

*The limitations and future prospects of this study are as follows: (1) This study employs a quantitative approach to investigate the impact of openness in Boundary Shell systems on the sustainable development of high-tech industries, which may not fully capture the complex and multifaceted policy implications present in today's intricate realities. While this method has its merits, it is limited in providing a comprehensive understanding of the nuanced dynamics at play. To address this limitation, future research could incorporate qualitative methods to gain deeper insights into the complexity of policy impacts. (2)The concept of Boundary Shell systems in this study refers to the interconnected networks of actors, institutions, and policies that shape the development of high-tech industries. This theoretical framework provides a useful lens for examining the impact of openness on sustainable development in this context. However, it is important to recognize that there are alternative theories and perspectives that could offer additional insights into this complex issue. Future research could benefit from incorporating diverse theoretical perspectives to provide a more comprehensive understanding of the factors that influence sustainable development in high-tech industries.(3)The experimental data sample in this paper focuses solely on policy impacts within specific countries and regions, thereby limiting its applicability to other geographical contexts. In order to enhance the generalizability of the findings, future research should strive to expand its scope and include a broader range of countries and regions. By doing so, researchers can develop a more holistic understanding of policy impacts that transcend national boundaries and have greater significance for global decision-making.*

The complete set of modifications can be found by referring to page 25-29 of the manuscript.

We hope that you will find these changes satisfactory.Thank you again for your constructive feedback and suggestions.

**Question 7:**

It is necessary to further improve and refine the logical structure, theoretical framework, research methods, and results analysis section to enhance the quality and reliability of the paper.

**Response 7:**

Thank you for your constructive feedback and suggestions for improvement. We appreciate your time and effort in reviewing our manuscript.

We have carefully considered your comment regarding the need to further improve and refine various aspects of our paper, including the logical structure, theoretical framework, research methods, and results analysis section. In response to your suggestion, we have made the following revisions:

（1）We have reorganized the logical structure of the paper to ensure that the flow of ideas is clear and coherent. This includes a more detailed outline of the main sections and subsections, as well as a revised sequence of presentation to enhance the overall readability and comprehension of the manuscript.

（2）We have revisited and refined our theoretical framework to better align it with the research questions and objectives of our study.

（3）We have also reviewed and improved our research methods section to provide a more detailed description of the procedures used in our study, including any modifications or enhancements made during the data collection process.

（4）we have thoroughly reviewed and analyzed our results, providing a more detailed interpretation and discussion of the findings in the results analysis section. This includes a clearer presentation of the data, as well as a more robust discussion of the implications and significance of our findings for the field.

The specific modifications have been addressed in the previous response, and can also be found by referring to the red highlighted sections in the manuscript.

**Question 8:**

The paper includes seven funding projects. Are all these funding projects relevant to the research content?

**Response 8:**

We are deeply grateful for the meticulous review and invaluable feedback you have provided on our manuscript. After careful consideration, we have decided to reduce the support from one of the funding projects, as per your suggestion.

We acknowledge that the previously mentioned funding project may have been redundant and not entirely relevant to the focal point of our research. In the revised version of our paper, we will be more attentive to the core research elements and ensure that each funding project aligns closely with the objectives of our study.

We express our sincere appreciation for your guidance and resubmission of the modified manuscript for your further scrutiny. If you have any additional recommendations or comments, please do not hesitate to let us know.

In conclusion, we would like to express our sincere gratitude to you for your constructive comments and suggestions, which have helped us to significantly improve the quality and presentation of our paper.

**RESPONSE TO REVIEWER 2**

**Question 1:**

Lines 89-90 would benefit from including the initial source: Cao HX. Shell (Jieke) phenomenon and its scientific frame. Science and Technology Report No. 88005. Chinese Academy of Meteorological Science. SMA. 1988.

**Response 1:**

We would like to extend our heartfelt appreciation for the insightful feedback provided by the esteemed reviewers. In response to their constructive criticism, we have made several revisions to enhance the manuscript's quality. Specifically, we have removed any inappropriate statements and incorporated appropriate citations to bolster our arguments. The examples of modifications in manuscript are as follows(indicated in italics):

*In the field of intelligent science, the Boundary Shell theory is considered a fundamental theory in China. It provides a comprehensive framework for understanding and managing the exchange of energy, matter, and information between a system and its environment, with a particular emphasis on the role of Boundary Shells. The theory proposes that the Boundary Shell model can represent a system, encompassing 'system' (core), 'wall' (boundary), 'gate' (interface for exchange between the system and the environment), and 'environment' (external environment) [8].*

We have meticulously incorporated appropriate referencing throughout the manuscript to ensure precision and adherence to academic standards. We are deeply grateful for the reviewers' guidance and attention, and we firmly believe that these revisions have substantially enhanced the quality of our work.

The complete set of modifications can be found by referring to page 5 of the manuscript.

We hope that you will find these changes satisfactory.Thank you again for your constructive feedback and suggestions.

**Question 2:**

The functions of the boundary are indistinct and require clarification. The listed information such as “particularly the role of boundary shells in regulating and managing the exchange of matter, energy, and information” (lines 98-103), “maintaining a sustainable and healthy ecological environment, highlighting the need to consider the interconnectedness of ecological, economic, and social factors in sustainable development.” (lines 111-114), lacks precision and clarity.

**Response 2:**

We appreciate your insightful suggestion here.We have carefully considered your suggestions and made the necessary revisions to address the issues raised. The following is our response to your comments and the changes we have made to the manuscript:

**（1）Clarification of boundary shell functionality**

Regarding the clarification of boundary shell functionality, we have provided a detailed explanation of the role of boundary shells in regulating and managing the exchange of matter, energy, and information. To enhance the clarity of this concept, we have incorporated relevant literature and conducted a thorough analysis based on our research findings. Furthermore, we have emphasized the differences in boundary shell functionality across various scales and their applications in ecological, socio-economic, and other domains.

To better illustrate the operating mechanism of the boundary shell system, we have included an illustrative diagram (see attached figure) in the manuscript. This diagram summarizes the basic components and interaction processes of the boundary shell system, helping readers gain a more intuitive understanding of how boundary shell functionality is achieved.

**Fig 1.A Schematic diagram of the bound-shell structure.**

Through these revisions, we aim to better elucidate the concept of boundary shell functionality and its significance in practical applications.We will take them seriously and make the necessary modifications promptly to ensure the quality of our work.

The complete set of modifications can be found by referring to page 3-6, of the manuscript.

**（2）Revision of lines 98-103, lines 111-114 for precision and clarity**

Thank you for your feedback and for bringing the reference error to our attention. We apologize for any inconvenience this may have caused.

We have thoroughly reviewed our manuscript and can confirm that the reference source was indeed missing. We understand the importance of accurate referencing and take full responsibility for this oversight.To rectify this issue, we have updated our manuscript to include the missing reference source with the correct number. We have also carefully checked all other references to ensure their accuracy and completeness.

We apologize for any confusion or frustration this may have caused and appreciate your attention to detail in helping us improve our work.

The complete set of modifications can be found by referring to page 6, of the manuscript.

**Question 3:**

Figure 1, Figure 2: It is recommended that the authors explore the potential external or internal factors contributing to the observed fluctuations. The authors could speculate on it and provide further clarification in the Conclusion or Discussion section.

**Response 3:**

We express our gratitude for the insightful comments and suggestions provided by the reviewer. We acknowledge the value of exploring potential external or internal factors.The following is our response to your comments and the changes we have made to the manuscript:

**（1）Revised and Corrected two Figures**

In response to this comment, we have carefully considered the possible factors that may have influenced the results presented in Figure 1 and Figure 2. To address this issue, we have redrawn two figures to more clearly illustrate the fluctuation of the indicators and further clarified them in the Conclusion section.The redrawn figures are as follows:

**Fig5. The ratio of C1- C3 in 2011-2022**

**Fig6. Ratio of bounds between D1 and D2 from 2011 to 2022**

**（2）Exploring Potential External or Internal Factors Leading to Observed Fluctuations**

We appreciate your interest in our work and agree that exploring the potential external or internal factors contributing to the observed fluctuations would add value to our study.In response to your comment, we have carefully considered the possible factors that may have influenced the results presented in Figure 1 and Figure 2.We have discussed potential confounding factors in the revised manuscript. We have speculated on the possible influence of these factors on our findings and provided further clarification in the Conclusion or Discussion section.

The examples of modifications in manuscript are as follows(indicated in italics):

**① Exploration and discussion of Fig 1**

*This highlights the need for further investigation into the factors contributing to these fluctuations and potential measures to mitigate their impact on the overall performance of the system. It is crucial to analyze the external factors that may have influenced the fluctuations in the R&D capability boundary ratio. These could include changes in market conditions, advancements in technology, or shifts in consumer preferences. By identifying and understanding these external influences, organizations can better anticipate and adapt to the evolving landscape, ensuring that their R&D efforts remain aligned with market demands.*

*This revelation indicates that the Fig. 5 reveals that the ranges of fluctuation for the new product sales capability boundary ratio and new product development capability boundary ratio are quite similar, implying that the high-tech industries' capabilities in both areas are relatively evenly matched. However, the effective R&D capability boundary ratio exhibits a wider range of fluctuations, indicating a certain degree of imbalance between R&D investment and effectiveness. Therefore, there is an imperative need to strengthen R&D management and optimize the investment structure in this area, to enhance the efficiency and effectiveness of R&D endeavors.To achieve this, it is crucial to adopt a comprehensive approach that encomTo achieve this, it is crucial to adopt a comprehensive approach that encompasses various aspects of R&D management. Firstly, organizations must establish clear goals and objectives for their R&D activities, aligning them with the overall business strategy. This will help ensure that resources are allocated efficiently and effectively towards projects that have the highest potential for success.In addition to these measures,strengthening R&D management and optimizing investment structures are imperative for enhancing the efficiency and effectiveness of R&D endeavors. By establishing clear goals, fostering collaboration, investing in talent, engaging with external partners, and continuously monitoring performance, organizations can position themselves for sustained success in the rapidly evolving landscape of research and development.*

**② Exploration and discussion of Fig 2**

*The examples of modifications are as follows(indicated in italics):*

*The study reveals that the protective capabilities of the two boundary layers are not equal, and the innovation capability boundary layer exhibits a stronger ability to safeguard against potential threats or risks. The study findings highlight a significant disparity in the protective capabilities of the two boundary layers, with the innovation capability boundary layer demonstrating a markedly stronger ability to safeguard against potential threats or risks. This suggests that organizations must prioritize and invest in cultivating a culture of innovation if they aim to effectively navigate the complex and dynamic business landscape of today.Based on Fig.6, it can be observed that innovation plays a critical role in safeguarding organizations against potential threats and risks. By prioritizing innovation, fostering a culture of openness and collaboration, adopting a flexible approach, and investing in R&D efforts, organizations can strengthen their protective capabilities and position themselves for long-term success in an increasingly competitive business landscape.*

In addition, we also conducted an in-depth comparative analysis of the revisedIn addition, we also conducted an in-depth comparative analysis of the revised and original model of the boundary ratio, and the comparative diagram is as follows:

The complete set of modifications can be found by referring to page 19-25, of the manuscript.

**Question 4:**

Despite the thorough presentation of the results and conclusion, it is crucial to compare the current findings with other theories, including the “traditional model”. I urge the authors to either expand the Conclusion or include a Discussion section.

**Response 4:**

We have appreciated the reviewer's suggestion to compare our findings with other theories, including the traditional model. We agree that it is important to provide a comprehensive discussion of existing literature. Therefore, we have planned to expand the Conclusion section to include a comparison with the traditional model.

The examples of modifications in manuscript are as follows(indicated in italics):

# *5.Conclusions remarks and implication*

***5.1 Conclusions***

*Through a series of investigations conducted in this paper, we aim to explore whether the openness of Boundary Shell systems impacts the sustainable development of high-tech industries. Throughout this process, relevant data has been collected and subjected to meticulous analysis. In this section, we will provide a comprehensive summary and discussion of our research findings with the purpose of gaining a deeper understanding of the factors and underlying mechanisms that drive the sustainable development of high-tech industries through the lens of Boundary Shell theory. The main results are summarized as follows:*

(1) *This study delves into the complex interplay between the quantity of Boundary Shell and internal system.*
(2) *After the refinement of the boundary system ratio model, a significant shift in the prioritization of indicator industries has occurred.*

*(3)The boundary system ratio model has undergone a continuous refinement process to enhance its capacity in evaluating the sustainable development capabilities of high-tech industries.*

***5.2 Policy implication***

*In light of the aforementioned discoveries, the following research implications are suggested.*

(1) *Collaboration is the key to unlocking the full potential of high-tech industries and ensuring their long-term success.*

*(2) The principle of entropy equilibrium provides a valuable framework for evaluating the stability and lifespan of high-tech industry Boundary Shell systems.*

*(3) The openness of Boundary Shell systems plays a critical role in promoting the sustainable development of high-tech industries.*

***5.3. Limitations***

*The limitations and future prospects of this study are as follows: (1) This study employs a quantitative approach to investigate the impact of openness in Boundary Shell systems on the sustainable development of high-tech industries, which may not fully capture the complex and multifaceted policy implications present in today's intricate realities. While this method has its merits, it is limited in providing a comprehensive understanding of the nuanced dynamics at play. To address this limitation, future research could incorporate qualitative methods to gain deeper insights into the complexity of policy impacts. (2)The concept of Boundary Shell systems in this study refers to the interconnected networks of actors, institutions, and policies that shape the development of high-tech industries. This theoretical framework provides a useful lens for examining the impact of openness on sustainable development in this context. However, it is important to recognize that there are alternative theories and perspectives that could offer additional insights into this complex issue. Future research could benefit from incorporating diverse theoretical perspectives to provide a more comprehensive understanding of the factors that influence sustainable development in high-tech industries.(3)The experimental data sample in this paper focuses solely on policy impacts within specific countries and regions, thereby limiting its applicability to other geographical contexts. In order to enhance the generalizability of the findings, future research should strive to expand its scope and include a broader range of countries and regions. By doing so, researchers can develop a more holistic understanding of policy impacts that transcend national boundaries and have greater significance for global decision-making.*

The complete set of modifications can be found by referring to page 25-29 of the manuscript.

**Question 5:**

To ensure clarity, eliminate duplications such as lines 91-92 and 100-101 and correct all references typos found in lines 38, 43, 46, 47, and so on. Additionally, the Reference list should follow the guidelines of the journal and includes DOIs when applicable.

**Response 5:**

We appreciate the reviewer's suggestion to ensure clarity and eliminate duplications. We have made the necessary changes to our manuscript, including correcting typos in lines 38, 43, 46, 47, and so on. Additionally, we have revised the Reference list to follow the guidelines of the journal and include DOIs when applicable.

In conclusion, we would like to express our sincere gratitude to you for your constructive comments and suggestions, which have helped us to significantly improve the quality and presentation of our paper.
